# Robotic assisted treatment of flank hernias: case series

**DOI:** 10.1186/s12893-020-00843-3

**Published:** 2020-08-12

**Authors:** Matteo Di Giuseppe, Francesco Mongelli, Maria Marcantonio, Davide La Regina, Ramon Pini

**Affiliations:** 1 Department of Surgery, Ospedale Regionale di Bellinzona e Valli, via Ospedale 12, 6500 Bellinzona, Switzerland; 2 Department of Surgery, Ospedale Regionale di Lugano, via Tesserete 46, 6900 Lugano, Switzerland

**Keywords:** Robotic-assisted, Minimally invasive, Flank hernia, Abdominal wall, Mesh

## Abstract

**Background:**

Flank hernias are uncommon, surgical treatment is challenging and the minimally-invasive approach not always feasible. The aim of this study was to report the safety and feasibility of the robotic-assisted repair.

**Methods:**

The study was approved by the local ethic committee (2019–01132 CE3495). A retrospective search on a prospectively collected dataset including demographic and clinical records on robotic surgery at our institution was performed to identify patients treated for a flank hernia. Patients were followed-up 6 months.

**Results:**

From January 2018 to December 2019, out of 190 patients who underwent robotic-assisted hernia surgery, seven with incisional flank hernia were included. Median age was 69.0 years (IQR 63.2–78.0), BMI was 27.3 kg/m^2^ (IQR 25.8–32.3) and two patients were male (29%). All patients were referred to surgery because of pain, whereas one of them described recurrent episodes of small bowel obstruction.

The median hernia defect measured 25 mm ((IQR 21–40), median mesh diameter was 10 cm (IQR 10–12.5) and median operative time was 137 min (IQR 133–174). No intraoperative complication occurred.

Postoperatively, one patient developed a pneumonia, which required antibiotics. Length of hospital stay was 4.0 days (IQR 3.0–7.7). Six months after surgery, neither recurrence nor chronic pain were recorded.

**Conclusions:**

Robotics in abdominal wall hernia surgery remains a matter of debate, despite a growing interest from the surgical community. In our reported experience with flank hernias, we found the robotic-assisted approach to be safe and feasible for the treatment of this uncommon clinical entity.

## Background

Flank hernias are uncommon clinical entities, located lateral to the rectal sheath in the area 3 cm above and below the umbilicus [1]. Most commonly, these are incisional hernias, whereas spontaneous, post-traumatic and congenital forms have been described [2–4]. The surgical treatment is challenging, as a proper mesh fixation and overlap are made demanding by the regional bone and neurovascular structures, with consequent limitation in the safety and feasibility of the minimally invasive approach [2, 5–7].

In the literature, open repair of flank hernia has been described in many series. However, the retrospective nature of most studies and the overall sample exiguity limit the level of evidence [5]. The open approach has been shown to be effective, but postoperative complication rate and hospital stay remain an issue [2, 3, 8–11]. In our experience, the laparoscopic treatment of this kind of hernias is technically demanding and not always feasible, so that conversion to open surgery is often needed.

Robotic surgery has been applied to ventral hernia repair, though flank hernias are reported sporadically and a strong evidence is far to be found [12–14].

The aim of this study was to report about the safety and feasibility of flank hernias repair with a minimally invasive robotic-assisted approach.

## Methods

### Patients’ inclusion

Written non-opposition consents were administered to patients and the local ethical committee approved the study (Comitato Etico Cantonale Ticino n. 2019–01132 CE 3495). STROBE statement was applied [15].

At the Bellinzona Regional Hospital, Switzerland, a retrospective search on a prospectively collected dataset on robotic surgery over a 2-years period, from January 2018 to December 2019, was performed. We included all patients who were treated for an incisional flank hernia with a robotic-assisted approach. Flank hernias were defined according to the definition of Muysoms FE et al. [1]: lateral to the rectal sheath in the area 3 cm above and below the umbilicus but not located in the lumbal, iliac or subcostal regions. Patients were excluded in case of flank bulges, defined as a protrusion of the abdominal content without any interruption or defect of the abdominal fascia.

The dataset included demographic and clinical records such as age and sex, past medical history, hernia etiology, symptoms, defect side and larger diameter, mesh dimension, operative times, conversion rate, length of hospital stay, complications and recurrence rate at 6 months (Table 1). All operations were performed by the same two senior general surgeons. All patients were followed-up in our outpatient clinic at 1 and 6 months after surgery, were asked about chronic pain and underwent a clinical examination to rule out the presence of seroma or recurrence.

**Table 1:** Patients’ characteristics

Age (years)	Gender	Body mass index (kg/m^2^)	Side	Comorbidities	Etiology	Index operation	Symptoms	Larger hernia diameter (mm)	Mesh dimension (cm)	Operative time (min)	Hospital stay (days)	Postoperative complications
64	Female	25.4	Left	None	Trocar site hernia	Laparoscopy for gynecological disease	Pain	24	13 × 12	149	4	None
80	Female	27.3	Left	None	Flank mini-laparotomy (specimen extraction)	Laparoscopy for unknown benign tumor	Pain	25	10 × 10	135	10	None
63	Male	34.1	Right	Hypertension, diabetes, obesity	Trocar site hernia	Laparoscopic-assisted inguinal hernia repair	Pain	84	20 × 20	137	3	None
79	Female	27.2	Left	Pulmonary disease, cardiac history	Flank laparotomy	Iliac crest bone harvesting	Pain, small bowel occlusion	45	10 × 10	236	9	Pneumonia
56	Female	28.4	Left	Hypertension, diabetes	Trocar site hernia	Laparoscopic-assisted bilateral adnexectomy	Pain	15	10 × 10	133	3	None
75	Female	33.6	Left	Hypertension	Flank laparotomy	Unknown	Pain	25	12 × 12	115	4	None
69	Male	23.1	Left	None	Trocar site hernia	Laparoscopic-assisted inguinal hernia repair	Pain	20	9 × 6	182	2	None

Descriptive statistics were presented as absolute frequencies and percentage for categorical variables and median with interquartile ranges (IQR) for continuous variables.

### Operative technique

**Fig. 1:**
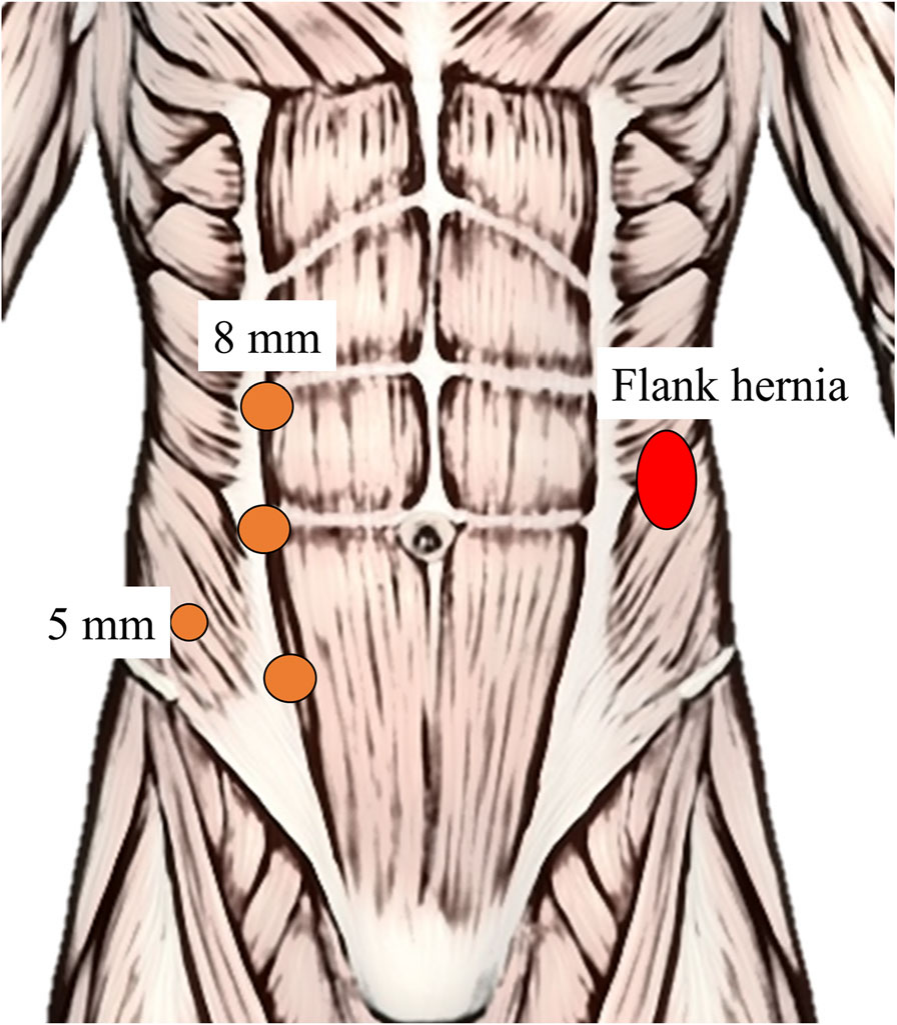
Trocar placement on the abdomen

The operations were performed in the supine position under general anesthesia with preoperative antibiotic therapy and antithrombotic devices. The pneumoperitoneum was created either inserting a Verres needle in the left subcostal region or with the open technique according to the past surgical history and surgeon’s preference. Three 8-mm robotic trocars were inserted along the pararectal line, opposite to the hernia site. One 5 mm assistant trocar was inserted between and lateral to two of the robotic trocars (Fig. 1). After the docking of the Da Vinci Xi robot system (Intuitive Surgical) we used tree robotic arms, firstly inserting the 30° camera through arm 2, then the fenestrated bipolar forceps through arm 1 and the monopolar curved scissors through arm 3.

**Fig. 2:**
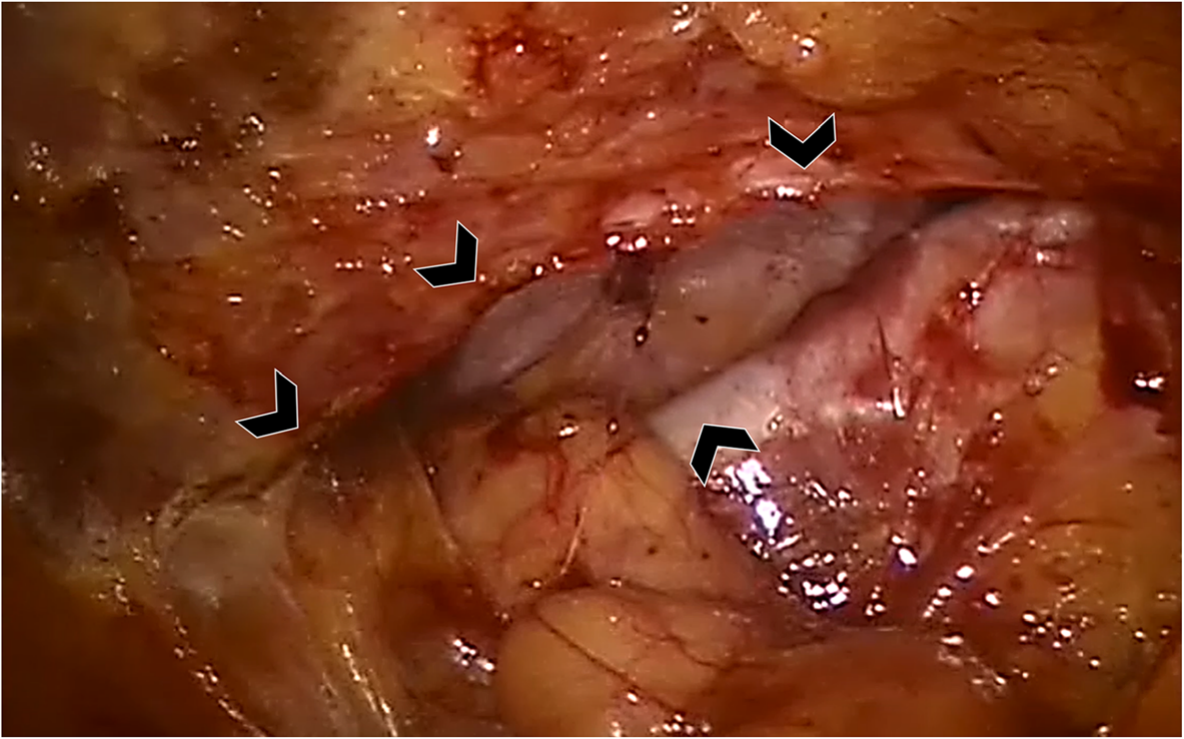
Intraoperative image showing the hernia defect and the prepared preperitoneal space

After careful dissection of the peritoneal adhesions, it is essential to incise the peritoneum at least 5 cm medially to the medial hernia margin, in order to create the preperitoneal space for a proper mesh overlap. To achieve this, a sterile measuring tape was used to measure the peritoneum incision line. The preperitoneal space was completely prepared and the hernia sack dissected from the subcutaneous tissue. The fine robotic assisted preperitoneal dissection can be performed taking care not to injure the peritoneum, also where it is typically very thin, i.e. posterior to rectus sheaths. After complete preparation of the preperitoneal space to guarantee a 5-cm mesh overlap on the hernia defect (Fig. 2), we inserted a suction Redon drain through the skin in order to prevent a postoperative seroma. Generally, the drain was left in place for 48 h after surgery.

After inserting the robotic needle holder through the arm 3, the hernia defect was closed with a continuous Covidien V-Loc-0 barbed suture, usually under a pneumoperitoneum pressure of 8 mmHg. Once closed the hernia defect, we used again the sterile measuring tape to exactly cut the mesh (Covidien Parietene) to the measured size and shape. The mesh fixation was achieved with several interrupted Vicryl 3–0 sutures. At the end, the peritoneum was sutured with a continuous Covidien V-Loc 4–0 barbed suture. After the robot undocking, the trocars were removed under vision and the fascia was not closed (Video 1).


**Additional file 1: Video 1.** Flank hernia repair with a robotic-assisted approach.

## Results

From January 2018 to December 2019, out of 190 patients who underwent robotic-assisted hernia surgery, 39 were treated for an incisional hernia, of whom seven consecutive patients with a flank hernia were included in the present study. Median age was 69.0 years (IQR 63.2–78.0), two patients were male (29%) and body mass index (BMI) was 27.3 kg/m^2^ (IQR 25.8–32.3). Three patients had hypertension (43%), one cardiac history (14%), two diabetes (29%) and one obesity (14%). All patients were referred to surgery because of pain, whereas one of them described recurrent episodes of small bowel obstruction. All hernias were incisional, three after a flank laparotomy and four trocar site hernias.

The median measure of the hernia defect was 25 mm (IQR 21–40), the median mesh larger diameter was 10 cm (IQR 10–12.5) and the median operative time was 137 min (IQR 133–174). No intraoperative complication occurred and no cases of conversion to open surgery were recorded.

Postoperatively, one patient affected from chronic obstructive pulmonary disease developed a pneumonia, which led to a longer hospital stay and was successfully treated with antibiotic therapy. The median length of hospital stay was 4.0 days (IQR 3.0–7.7). At 6 months after surgery all patients completed the follow-up and neither seroma/recurrence nor chronic pain were recorded.

## Discussion

Our study shows high successful rate of the robotic approach in flank hernia surgery, without any case of conversion to open surgery or presence of recurrence during a 6-month follow-up.

Hernias of the flank are an uncommon occurrence in everyday surgical practice. According to the European Hernia Society, in flank hernias the fascial defect is localized lateral to the rectal sheath in the area 3 cm above and below the umbilicus [1]. Such classification clearly distinguishes these hernias from the iliac and subcostal ones, as well as from flank bulges. The latter, in fact, occurs in absence of an obvious fascial defect and should not be understood as a hernia, but as a denervation injury of the musculoaponeurotic layers of the flank [6]. Almost all flank hernias occur after a surgical operation either laparoscopic or open, even if other causes are occasionally reported [2–4]. After a flank incision, hernias can occur in up to 17% of patients. Known risk factors are the incision length, the presence of metabolic syndrome, smoke, heart, lung and renal diseases [5].

The indication to surgically treat a flank hernia is clear in symptomatic cases, as an intermediate risk of complications, such as incarceration and strangulation, has been reported [7]. The treatment strategy depends on several factors but, anyhow, it comes down to challenging operations. In fact, the regional bone and neurovascular structures limit the safety and feasibility of mesh fixation and overlap [2]. In addition, due to the rarity of such surgical condition, no standardized technique can be suggested. Several approaches have been described in the literature and, in the minimally invasive era, the laparoscopic repair with mesh placement whether intraperitoneal or preperitoneal seems to represent a valid treatment option [2, 9, 10, 16–21].

In our previous experience of laparoscopic flank hernia repair, we encountered technical difficulties during the dissection of the preperitoneal space, as the thin peritoneal layer may break or even the posterior rectus sheath can be injured. In addition, a closure of the fascial defect is rarely achievable with laparoscopy. Since 2017, in our Department of General Surgery, the treatment of abdominal wall hernias is performed with a robotic-assisted minimally invasive approach, using the da Vinci Xi system (Intuitive Surgical). The da Vinci system offers a 30° - 3D camera with magnificent visualization of the operative field. Its technology provides for high precision movements. Thanks to the EndoWrist technology-related instruments’ range of motion, the da Vinci system allows precise dissecting and suturing in narrow spaces, in our opinion hardly achievable with other minimally invasive techniques. Therefore, according to our experience, in minimally invasive hernia surgery, preperitoneal dissection, components separation, fascial defect closure and mesh fixation are properly to perform and, in complex cases, better achievable with robot-assisted surgery [22].

From January 2018 to July 2019, we performed 7 consecutive robot-assisted preperitoneal repair of flank hernias. None of the operations had to be converted to open surgery. We systematically treated patients with hernia defect closure and 5 cm mesh overlapping in order to minimize seroma, bulging and recurrence, following the recommendations of the International Endo Hernia Society (IEHS) [23]. Furthermore, we decided to include only patients with incisional hernias, as treatment and outcomes in case of congenital defect may vary and represent a source of bias [24].

In a series of 22 patients affected from lateral abdominal wall hernias and treated with open surgery, Cavalli M et al. [8] reported an early complication rate of 9.1% (two cases of hematoma), a mean length of hospital stay of 4.8 days and one case of recurrence after a mean follow-up of 44 months. In the literature dealing with open treatment of flank hernias, the reported complication rate varies from 3 to 42% and the recurrence rate from 0 to 11% [2, 3, 9–11]. Edwards C et al. [7] reported on 27 patients with flank hernias operated laparoscopically. The length of hospital stay was 3.1 days. No recurrence was seen after 4 months follow-up. However, 3 patients had chronic postoperative pain, presumably secondary to mesh fixation or compression of nerve structures. Novitsky YW et al. [25] described excellent results of laparoscopic treatment of flank hernias in 14 patients with short hospital stays and no recurrence after 35 months. However, all hernias were of traumatic origin. Interestingly, the authors themselves commented how robot-assisted surgery may facilitate the minimally-invasive repair of flank hernias and extend the minimal access benefits to hernias that are commonly treated with open surgery.

In our series, no conversion to open surgery was necessary. Median length of hospital stay was 4 days and one patient with medical history of severe chronic obstructive pulmonary disease developed a postoperative pneumonia that required a longer hospital stay, physiotherapy and antibiotic therapy.

This study has several limitations. Its nature does not include a comparison group and, due to its retrospective design, the data were not measured in a standardized way, with inherent measurement bias. In addition, a small number of cases was included, as flank hernias represent an uncommon surgical occurrence. For those cases, we have no data about a long-term follow-up.

Furthermore, the use of robots in general surgery remains controversial, also when the general surgical community appears to show a growing interest in robot-assisted procedures despite lack of evidence and increase of costs [22]. Before our robotic experience, we observed in the laparoscopic treatment of this kind of hernia a significant rate of conversion to open surgery because of several reasons: difficulties in the creation of the preperitoneal space with multiple peritoneal defects, unachievable fascial closure, impossible or too risky mesh fixation due to regional bone and neurovascular structures. In all patients we converted to open surgery, the hospital stay was significantly longer and, as a result of this, the in-hospital costs were consistently higher. In our series, none of the seven operations had to be converted to open surgery. Consequently, five on seven patients had short hospital stays. In this sense, we feel that a reduced length of stay may offset the costs of robotic surgery. However, in the current literature, there are only a few data, and none dealing with abdominal hernias, supporting our assertion [26, 27].

## Conclusions

Robotics in abdominal wall hernia surgery remains a matter of debate, despite a growing interest from the surgical community. In our reported experience with flank hernias, we found the robotic-assisted approach to be safe and feasible for the treatment of this uncommon clinical entity. Larger studies are needed to confirm our initial experience.

## Data Availability

The datasets used and/or analysed during the current study are available from the corresponding author on reasonable request.

## Abbreviations

IQR: Interquartile ranges
BMI: Body mass index.
